# The Immersive Virtual Reality Lab: Possibilities for Remote Experimental Manipulations of Autonomic Activity on a Large Scale

**DOI:** 10.3389/fnins.2018.00305

**Published:** 2018-05-08

**Authors:** Joshua Juvrud, Gustaf Gredebäck, Fredrik Åhs, Nils Lerin, Pär Nyström, Granit Kastrati, Jörgen Rosén

**Affiliations:** ^1^Department of Psychology, Uppsala University, Uppsala, Sweden; ^2^Goodbye Kansas Studios, Uppsala, Sweden

**Keywords:** virtual reality, eye tracking, pupil dilation, SCR, autonomic response

## Abstract

There is a need for large-scale remote data collection in a controlled environment, and the in-home availability of virtual reality (VR) and the commercial availability of eye tracking for VR present unique and exciting opportunities for researchers. We propose and provide a proof-of-concept assessment of a robust system for large-scale in-home testing using consumer products that combines psychophysiological measures and VR, here referred to as a Virtual Lab. For the first time, this method is validated by correlating autonomic responses, skin conductance response (SCR), and pupillary dilation, in response to a spider, a beetle, and a ball using commercially available VR. Participants demonstrated greater SCR and pupillary responses to the spider, and the effect was dependent on the proximity of the stimuli to the participant, with a stronger response when the spider was close to the virtual self. We replicated these effects across two experiments and in separate physical room contexts to mimic variability in home environment. Together, these findings demonstrate the utility of pupil dilation as a marker of autonomic arousal and the feasibility to assess this in commercially available VR hardware and support a robust Virtual Lab tool for massive remote testing.

## Introduction

Virtual Reality (VR) is defined as “an advanced form of human–computer interface that allows the user to interact with and become immersed in a computer-generated environment in a naturalistic fashion” (Schultheis and Rizzo, 2001). Unlike lab scenarios, video stimuli, or even augmented reality, VR is unique in that it is at the furthest end of the reality continuum (Milgram and Kishino, 1994) replacing real-world environments with virtual contexts. This allows for levels of stimulus control that surpass lab testing, absolute control of colors, textures, and luminance (Riva et al., 2016). The addition of integrated eye tracking, which is currently available to control interfaces and guide avatars in games (e.g., FOVE Eye Tracking VR Headset), opens up for measuring psychophysiological responses remotely on a large scale. In their extensive review of the VR literature, Lindner et al. (2017) concluded that eye tracking is becoming an important new technology in commercially available VR. The widespread use of VR by the public, in research, and in therapy is creating a need for more high-quality empirical studies examining VR and its capability for naturalistic “Big Data.”

In this paper, we propose and provide a proof-of-concept assessment of a robust system for large-scale in-home testing using consumer products that combine psychophysiological measures and VR, here referred to as a Virtual Lab. Specifically, our first aim is to simultaneously test and correlate two autonomic measures: skin conductance response (SCR), a well-established autonomic measure that has been reliably used in previous VR studies, and pupil dilation, a measure which has been demonstrated as a reliable autonomic measure but has yet to be tested and validated in VR. Our second aim is to demonstrate that these measures can be reliably recorded independent of physical location, demonstrating possibilities for remote testing. For a Virtual Lab to be a feasible reality in scientific research, it is important to establish that: (a) there is a demand for remote data collection on a large scale, (b) there is a wide availability of VR equipment in homes, and (c) there is a way to measure autonomic responses in a reliable and robust manner through the VR device. Each of which will be discussed briefly in the following sections.

### Remote data collection

Researchers across scientific fields have, to a large degree, relied on lab testing, which offers good control; however, it is difficult to use in/with remote demographics when collecting large samples. There is a demand for the availability of remote testing (eye tracking on tablet devices, Holland and Komogortsev, 2012; mobile eye tracking, Bulling and Gellersen, 2010; mobile phone testing, Tomlinson et al., 2009), but these efforts have been unable to fully bring a controlled testing environment to subjects remotely. As part of an effort to collect more data easily, many researchers have adopted massive home testing in the form of online testing and crowd sourcing systems, such as survey websites and Amazon MTurk (Buhrmester et al., 2011), which take advantage of online infrastructures to reach as many users as possible (the average MTurk study can reach approximately 7,300 participants; Stewart et al., 2015) at very low costs. Remote online platforms like MTurk, however, have clear weaknesses when it comes to the generalizability and reliability of responses (Goodman et al., 2013; Landers and Behrend, 2015) and lack experimental control and the richness of physiological measures. It has been proposed that VR offers the potential for self-help applications and remote therapy for patients who suffer from anxiety disorders; this collection of naturalistic data may further inform learning theory and behavioral therapy (Lindner et al., 2017).

### In-home availability and viability

The immersion and accessibility of VR has already been applied in many experiments and labs around the world. VR has been used to experimentally examine autonomic responses to social, proximal, and conditional threat (Rosén et al., 2017), and a recent review of 27 meta-analyses and systematic reviews (Riva et al., 2016) demonstrate that VR has been used in a large array of clinical settings (including anxiety disorders, Gorini and Riva, 2008; stress-related disorders, Botella et al., 2015; phobias, Parsons and Rizzo, 2008; panic disorders, Opriş et al., 2012; addiction, Hone-Blanchet et al., 2014; body-image disorders, Ferrer-García and Gutiérrez-Maldonado, 2012; autistic spectrum disorder, Aresti-Bartolome and Garcia-Zapirain, 2014). To date, the dependent variable being assessed in these studies are not derived from the VR device but measured outside of VR by external devices (e.g., Slater et al., 2010), clinical assessment (e.g., Carlin et al., 1997), or self-report surveys (e.g., Botella et al., 1998) requiring the participants to perform these studies in a lab with trained experimenters. During the last decade, however, VR has expanded outside scientific environments and has shown a quick growth into a mainstream tool in the average consumer's home (according to CCS Insight, a projected 24 million devices by 2018; Lamkin, 2016); further, it is a viable consumer-ready device that allows users to become fully immersed in a realistic, interactive 3D-world (e.g., Occulus Rift, HTC Vive, Sony Playstation VR; Aczél, 2016).

### Reliable autonomic measures

Psychophysiological measures can be used to assess the perceptual, cognitive, and emotional processes produced by physical stimuli. One standard tool is SCR, which measures autonomic activity in the nervous system and can be used to quantify response levels to stimuli. The reliability of SCR has been demonstrated in a plethora of previous studies (see for example Teghtsoonian and Frost, 1982; Lang et al., 1993; Löw et al., 2008). This method is, however, not available in people's homes and would practically be very difficult to measure remotely. Major hardware developers are moving toward an integration of eye tracking technology in commercially available VR headsets (e.g., FOVE Eye Tracking VR Headset, Tobii's VR4 for Vive Kit, SMI's HMD Eye Tracking for VR). For the last 10 years, eye tracking has been used increasingly to assess psychological mechanisms with notable accuracy (see Duchowski, 2007). Eye tracking (for review, see Trillenberg et al., 2004; Karatekin, 2007; Luna et al., 2008; Gredebäck et al., 2009) allows the measurement of pupil dilation, which is thought to index autonomic activity (Laeng et al., 2012). Pupil dilations and SCR are held to be comparable responses in the peripheral nervous system that are controlled by similar areas in the brain stem, such as the locus coeruleus (Laeng et al., 2012), and have previously been correlated with one another (Bradley et al., 2008; Wieser et al., 2009), although never in a VR environment.

#### Summary

While the demand for remote data collection and the in-home availability and reliability of VR are quite well established, the demonstration of reliable autonomic measures in VR is still lacking. Online platforms are capable of collecting data inexpensively and rapidly, but the level of experimental control and richness of the data collected through additional physiological measures can be drastically improved through VR. Eye-tracking measures are now commercially available in mobile VR headsets and provide access to physiologically measures remotely. To our knowledge, there have been no published studies that aim to assess the usability of integrating eye tracking within a virtual environment using VR, and no studies simultaneously measuring the pupillary response and SCR to VR stimuli. In his critical review of pupil methodology and measures, Aslin (2012) concluded with the statement “A final step in this process may, one day, be the use of virtual reality displays […] which would enable the experimenter to control the visual world” (p. 138). In the current study, we aim to establish pupil dilation as a reliable and robust autonomic measure in VR.

#### Current aims

As stated earlier, we believe that the time is right to integrate VR with home testing and use pupil dilations to gain reliable psychophysiological measures on large groups of participants, creating the foundation for a Virtual Lab. To assess the validity of these claims, three steps need to be taken. (1) We need to establish that we can effectively and reliably measure both SCR responses and pupil dilations in a single VR-based paradigm; (2) these two dependent measures further need to be correlated; and (3) the responses measured by pupil dilation and skin conductance are independent of the physical locale but responsive to the experimental manipulations in the VR environment (i.e., reliable remote testing should be independent of the physical environment in order to control for contextual confounds). The following aims of the current study address each of these steps to empirically test the utility of a Virtual Lab.

To complete these steps, we measured autonomic responses to spiders when compared to balls and beetles using pupil dilation and SCRs in a VR setting. Unlike previous studies demonstrating a fear response to spiders via stimuli presented on a computer monitor (Miltner et al., 2005; Rinck et al., 2005; Rinck and Becker, 2006; Gerdes et al., 2008), a Virtual Lab allows the physical distance of the spider to be manipulated in a standardized way (near or far from the participant) offering a high degree of control over stimuli presentations. We have previously shown the robust increase in SCR to proximal objects displayed in immersive virtual reality (Rosén et al., 2017). Additionally, half of the participants were immersed in a photo-realistic virtual environment of the exact same real-world laboratory room that they were physically seated in. The other half of the participants were physically seated in another entirely different room. This allowed us to test whether physical locale of the experiment influenced autonomic responses, an assumption critical for remote testing to be feasible. We then aimed to replicate the results of the first experiment with a new group of participants and with additional beetle stimuli in order to assess robustness and reliability.

## Methods

### Experiment 1

#### Participants

Forty adults (22 females; mean age 24.63 years) participated in Experiment 1. Four of the participants were excluded from the pupil task in the final analysis due to insufficient data across trials (more than 30% data loss), three participants were excluded from the SCR task because more than 10% of the trials were lost, and an additional six participants did not have SCR data recorded. The final sample was 36 adults (19 females; mean age 25.04) for the pupil task and 31 adults for the SCR task (16 females; mean age 26.40). In order to participate, all participants provided informed consent and received a 10€ gift voucher. This research was supported by a grant from the Swedish Research Council. The study was conducted in accordance with the standards specified in the 1964 Declaration of Helsinki and approved by the local ethics committee.

#### Materials

We used an HTC Vive VR headset (www.vive.com), which has a display resolution of 2,160 × 1,200 (1,080 × 1,200 per eye), 90 Hz refresh rate, and 110 degrees field of view. The VR headset had a built-in Tobii Glasses 2 eye-tracker (www.tobii.com/tech/products/vr/), with 100-Hz gaze sampling and absolute pupil measurement. The experiment was run in the Unity game engine on a lab PC capable of running 3D graphics.

#### Stimuli

**Figure 1:**
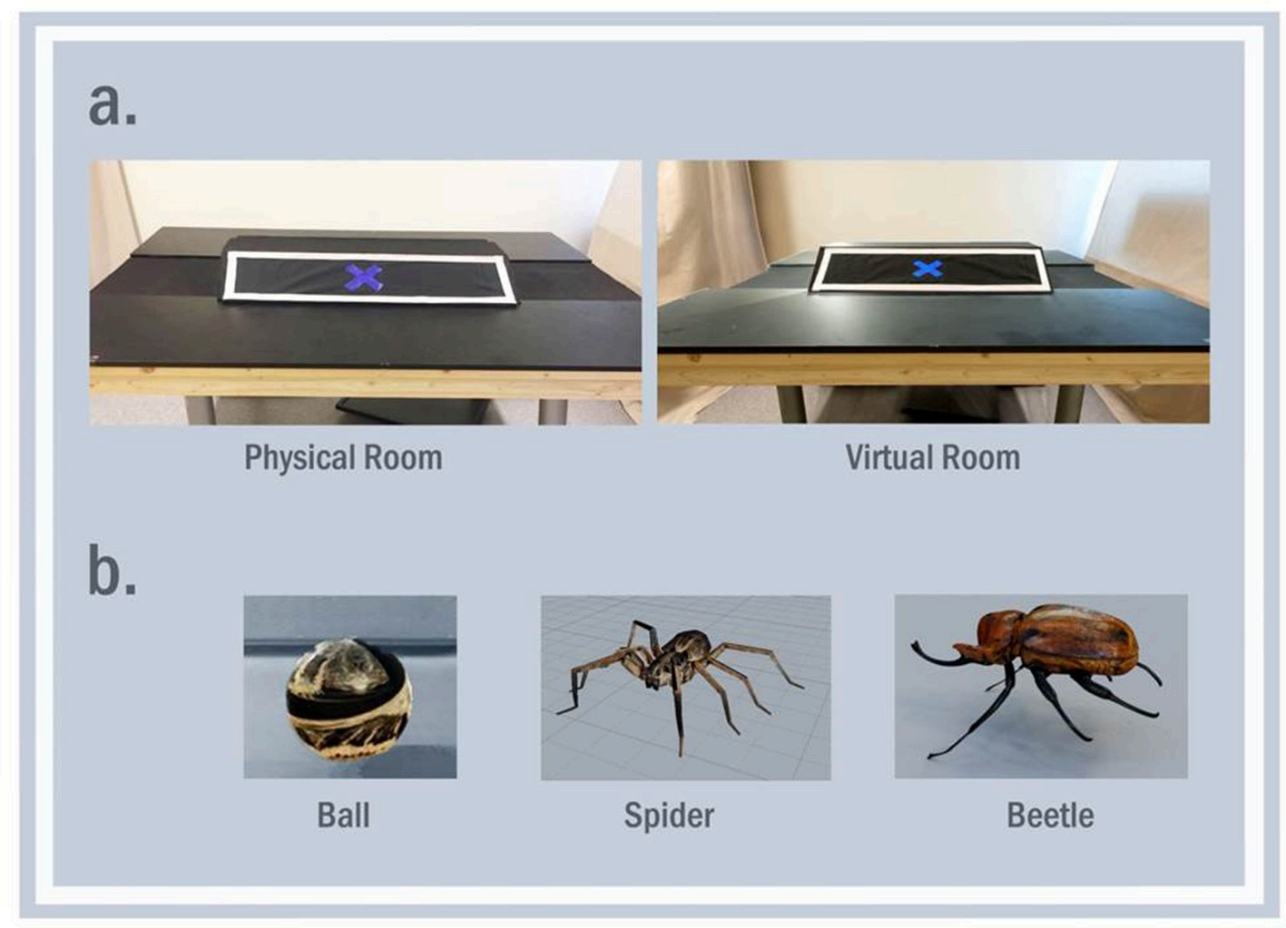
Side-by-side comparison of the real and virtual environment **(a)** and examples of the stimuli **(b)**, spider and ball (Experiment 1) and beetle stimuli (Experiment 2). Note that the images do not accurately reflect the color, luminance, and dynamic animations in the actual experiment.

The stimuli and environment were digitally created by Goodbye Kansas Studios (http://goodbyekansasstudios.com/) using Maya (www.autodesk.com/products/maya/overview) and Mudbox (www.autodesk.com/products/mudbox/overview). The environment was a lab room that was set up identically in both the virtual and real environment (see Figure 1). The virtual environment was created using photogrammetry, a method that uses 3D scan technology and photography of real physical environments to create a photorealistic effect to the graphics. The properties of the virtual environment space were set in Unity to reflect real-world proportional units measured in meters.

The stimuli were digitally created blue and brown spiders and balls (see Figure 1), approximately 30 × 30 cm. Stimuli appeared and moved toward the participant across three phases. During the beginning of the first appearance (distance of 60 cm), the stimuli were approximately 28 visual degrees; during the far approach (distance of 30 cm), the stimuli was approximately 53 visual degrees, and approximately 113 visual degrees, during the near approach (distance of less than 10 cm). To avoid differences in pupil dilation due to color, the exact same texture from the spider was applied to the balls. Both the spiders and the balls had an animation for their movement (walking for spiders, forward momentum for ball), and an additional idle animation for the spider for added realism.

#### Procedure

**Figure 2:**
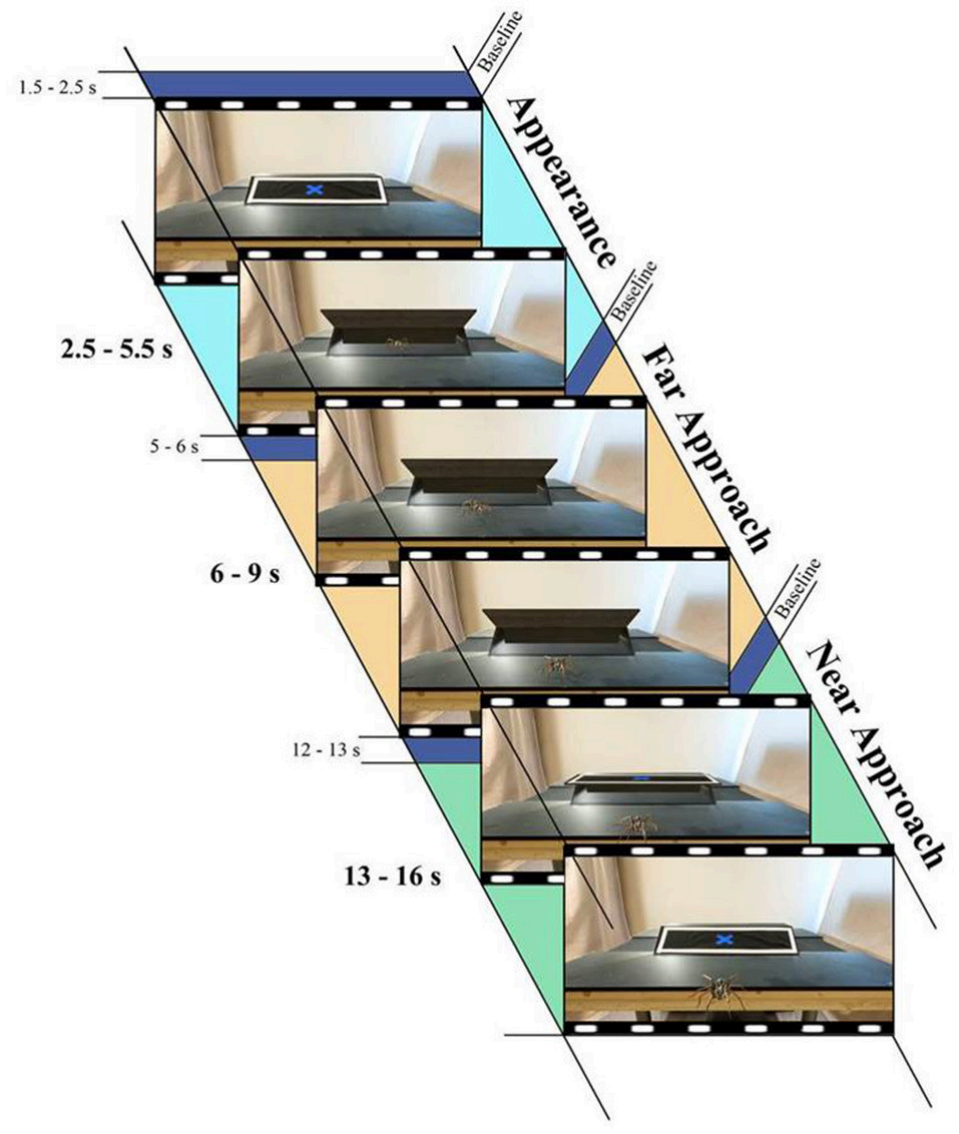
Time series illustrating the three distance periods and each corresponding baseline for pupil analyses. Time intervals were the same across all trials and conditions.

Participants arrived to one of the two experiment rooms: the same room as the virtual environment or a different room. In both conditions, participants were seated in front of a table and the experimenter sat behind the participant out of view. Participants were fitted with the VR headset and SCR Ag/CI electrodes. Prior to testing, participants were first “placed” in the VR room environment for approximately 2 min in order to allow participants to adjust to the VR experience, as well as to simultaneously calibrate the eye-tracker using an automatic one-point calibration procedure to ensure proper recording of pupil data. In the virtual environment, there was a black box with a front facing flap on the table in front of the participant (approximately 120 cm from the participant) with a blue “X” in the center. The experimenter instructed the participant to remain seated and to remain looking at the blue “X” throughout the experiment. During the experiment, the flap on the box would open revealing either a ball or spider inside (hereafter “First Appearance”). After 2.5 s, the stimuli would move forward and stop approximately 30 cm from the participant (“Far Approach”). After standing idle for 1 s, the stimuli continued moving toward the participant (“Near Approach”) until it “fell” off the table and appeared to fall onto the participants' lap, while the lid of the box simultaneously closed. After a 4-s pause, the box would re-open and a new trial would begin. A total of 16 trials were presented (8 spider trials, 8 ball trials) with the stimuli randomized and counter-balanced across participants. Refer Figure 2 for the timing of each trial. The total time of the experiment was 12 min.

### Recording and analysis

#### Pupil dilation

The dependent variable for each trial was the difference between mean pupil size during the three distance test periods (First Appearance, Far Approach, and Near Approach) and mean pupil size during the baseline period. The baseline period began 1,000 ms prior to the onset of each test period (see Figure 2). All trials were visually inspected for normal pupillary light reflex response. The analysis was performed in the open-source analysis program TimeStudio version 3.03 running in Matlab version 7.12 (www.timestudioproject.com; Nyström et al., 2016). We used a moving-average filter and gap interpolation across all data. The actual analysis, settings, and source code used for analyzing the data can be downloaded with uwid ts-674-f5e from the TimeStudio interface. We conducted a repeated measures ANOVA to examine pupil dilations to the different stimuli, at varying distances, and in the same or different physical room.

Due to concerns about the maximum mydriasis and miosis of the pupil while wearing a headset, and because this is one of the first studies to examine the pupil in VR, we checked that the baseline values of the pupil were within normal range (human pupil ranges in size from 7.5 to 8 mm at full mydriasis to 1.5–2 mm at full miosis, Alexandridis et al., 1985). We found that at across all baseline periods, there was an average baseline size of 3.73 mm (range of 3.55–3.87 mm) for all subjects, values comparable to other studies that have reported baseline pupil diameter (Privitera et al., 2010). We additionally performed a linear mixed model on the Wilkinson form to see whether baselines increased or decreased over time. There was a significant decrease in baseline values over trials (main effect of trial, estimate: −0.014287 mm/trial, *p* = 0.004), which suggests that the baseline returned after each trial, and also decreased a bit more. We interpret this as participants are more aroused during the experimental situation in the beginning of the session and then become habituated to the situation over time. Importantly, we did not find any difference in baseline values between conditions (*p* = 0.940).

#### Skin conductance responses

Skin conductance was recorded using the MP-150 BIOPAC system (BIOPAC Systems, Goleta, CA). Pre-gelled Ag/AgCl electrodes were placed on the left hand's palmar surface. The SCR signal passed through a high-pass hardware filter of 0.05 Hz and was analyzed with the Ledalab software package (Benedek and Kaernbach, 2010) implemented in Matlab (Mathworks Inc., Natick, MA). SCR was scored using the maximum phasic driver amplitude (Max.SCR) 1–4 s after each test period (see Figure 2) and then transformed (square root) and range corrected so that SCRs ranged from 0 to 1 (Lykken, 1972).

#### Data analyses

We had two within-subjects independent variables of interest (Condition and Distance), and one between-subjects independent variable (Room). The outcome measures of interest included mean pupil changes and SCRs to the stimuli presentations. Separate repeated measures ANOVAs with Greenhouse-Geisser correction were used for the pupil and SCR measures to study the interaction between Condition, Distance, and Room, and paired *t*-tests were used to test the comparisons. Means and standard deviations are reported in Table 1. Our primary interest was the autonomic response in the near approach based on the previous findings that indicate increased autonomic response to proximal stimuli when compared to distant ones (Rosén et al., 2017); therefore, only those results are reported here. The results for the appearance and far approach can be found in the Supplementary Materials.

**Table 1:** Mean score (SD) at each distance (appearance, far approach, near approach) and stimuli condition (spider, ball) for the pupil and SCR measures in Experiment 1.

		***n***	**Appearance**	**Far approach**	**Near approach**
Pupil	Spider	36	0.206 (0.099)	0.013 (0.082)	−0.147 (0.181)
	Ball	36	0.199 (0.102)	0.043 (0.096)	−0.295 (0.199)
SCR	Spider	31	0.371 (0.121)	0.371 (0.139)	0.410 (0.144)
	Ball	31	0.301 (0.130)	0.350 (0.129)	0.358 (0.132)

### Experiment 2

We aimed to replicate and extend the findings from Experiment 1 in a separate experiment with a new sample and using new additional stimuli in order to assess robustness and reliability.

#### Participants

Eighteen adults participated (11 females; mean age 26.88 years). Three of the participants were excluded from the pupil task in the final analysis due to insufficient data across trials (more than 30% data loss) and one participant was excluded from the SCR due to incomplete data (more than 10% of the trials were missing). The final sample was 14 adults (9 females; mean age 25.78) for the pupil task and 16 adults for the SCR task (10 females; mean age 25). (Participants provided informed consent and received a 10€ gift voucher for participating).

#### Stimuli

The stimuli and environment were identical to Experiment 1, with the addition of a beetle stimulus. The beetle was a digitally created blue and brown beetle (see Figure 1), approximately 30 × 30 cm. The distances and visual degrees of the stimuli were the same as those used in Experiment 1. The spiders, balls, and beetles had an animation for their movement (walking for spiders and beetles and forward momentum for ball), and an additional idle animation for the spider and beetle. The size of the spider and beetle and the walking and idle animations were identical.

#### Procedure

The general procedure was identical to Experiment 1, in which trials including a beetle were added and with no room condition. A total of 18 trials were presented (6 spider trials, 6 ball trials, and 6 beetle trials) with the stimuli randomized and counter-balanced across participants. Refer Figure 2 for the timing of each trial. In total, the experiment took approximately 15 min.

#### Recording and analysis

Recording and analysis measures and procedures were identical to Experiment 1. Means and standard deviations are reported in Table 2.

**Table 2:** Mean score (SD) at each distance (appearance, far approach, near approach) and stimuli condition (spider, beetle, ball) for the pupil and SCR measures in Experiment 2.

		***n***	**Appearance**	**Far approach**	**Near approach**
Pupil	Spider	14	0.218 (0.153)	0.047 (0.063)	−0.177 (0.213)
	Beetle	14	0.191 (0.109)	0.022 (0.054)	−0.237 (0.054)
	Ball	14	0.207 (0.113)	0.022 (0.101)	−0.364 (0.184)
SCR	Spider	16	0.420 (0.119)	0.362 (0.111)	0.481 (0.132)
	Beetle	16	0.376 (0.099)	0.368 (0.093)	0.433 (0.096)
	Ball	16	0.340 (0.119)	0.303 (117)	0.327 (0.078)

## Results

### Results from experiment 1

#### Pupil dilation

**Figure 3:**
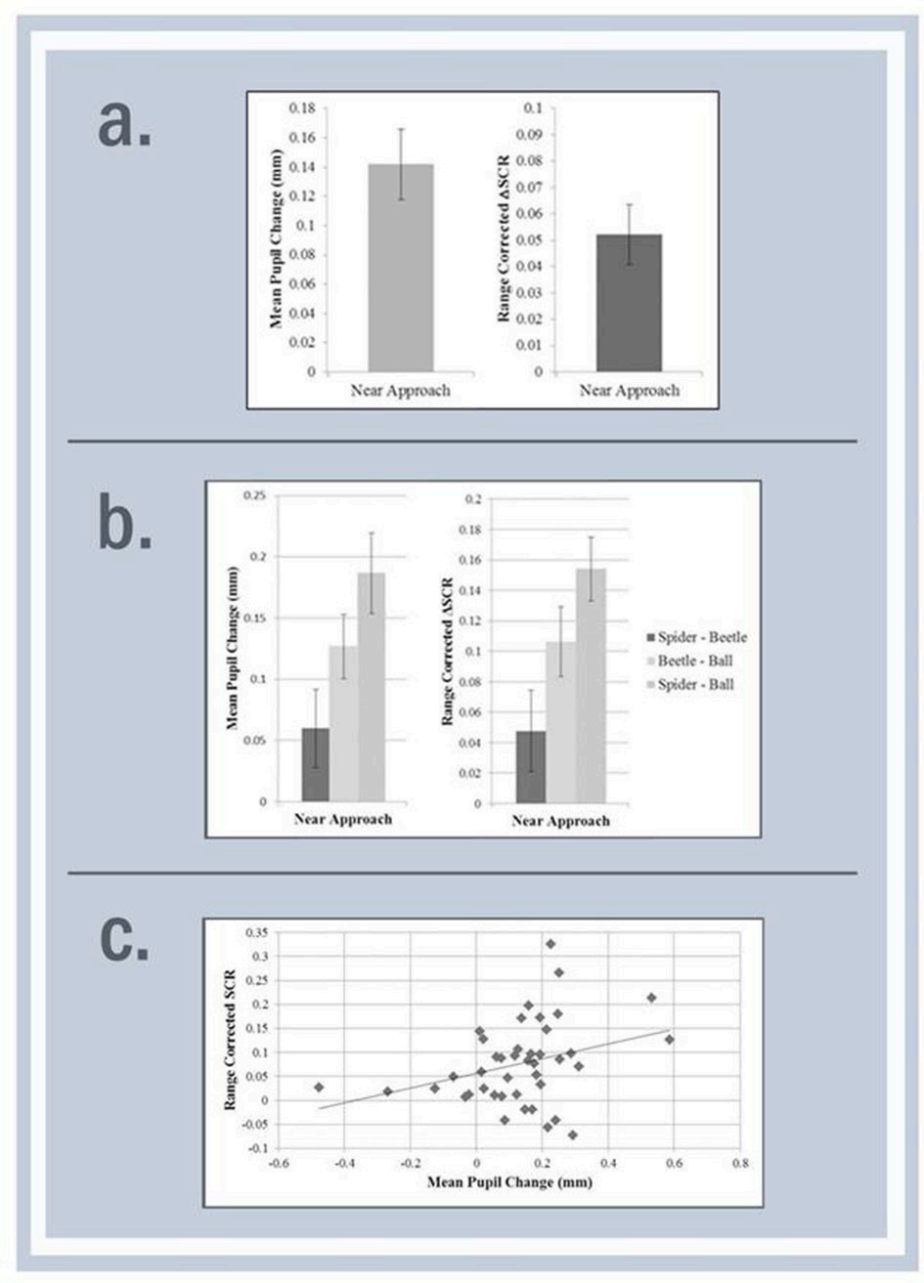
Mean difference scores for the near approach comparing spider vs. ball for pupil dilation and SCR measures in Experiment 1 **(a)**, spider vs. ball vs. beetle in experiment 2 **(b)**, and scatterplot with the aggregated data from experiments 1 and 2 showing a significant correlation between the difference scores form the spider and ball during the near approach **(c)**.

Results from the repeated measures ANOVA are shown in Table 3. There was a significant main effect of Condition where mean pupil dilation significantly differed between the stimuli, with significantly greater pupil dilation to the spider than to the ball *t*_(35)_ = 2.87, *p* = 0.007, *d* = 0.97. There was also a significant main effect of Distance whereby there was overall greater pupil dilation in the Near Approach than Appearance, *t*_(35)_ = −10.48, < 0.001, *d* = 3.54, and Far Approach *t*_(34)_ = −6.83, *p* < 0.001, *d* = 2.34. There was a significant interaction effect between Condition and Distance, meaning the pattern of pupil dilation in each condition differed at each distance (see Figure 3a). When examining the Near Approach, there was significantly greater pupil dilation to the spider than to the ball, *t*_(34)_ = 5.91, *p* < 0.001, *d* = 2.03. These changes in pupil size are comparable to significant changes observed in similar studies with both infants and adults (Gredebäck and Melinder, 2011; Hoehl et al., 2017; Hellmer et al., 2018).

**Table 3:** Results from the repeated measures ANOVAs with Greenhouse-Geisser correction for the pupil and SCR measures in Experiment 1 and Experiment 2.

	**Pupil Dilation**				**SCR**			
	**df**	**η2**	***F***	***p***	**df**	**η2**	***F***	***p***
**EXPERIMENT 1**								
Condition	28	0.30	12.13	<0.002	29	0.48	27.23	<0.001
Distance	39.92	0.68	60.49	<0.001	53.38	0.10	3.31	0.048
Condition × Distance	48.35	0.44	15.08	<0.001	47.25	0.11	3.40	0.051
**EXPERIMENT 2**								
Condition	19.90	0.63	12.28	<0.001	20.34	0.56	16.84	<0.001
Distance	14.06	0.75	34.65	<0.001	24.49	0.18	4.24	0.033
Condition × Distance	26.90	0.47	6.18	0.004	3.49	0.18	2.10	0.101

The main effect of Room was not significant (*p* = 0.704) and neither were the between-subjects interactions of Room with Condition and Distance (*p*s > 0.05), meaning that the pattern of pupil dilation between conditions and distance did not depend on the physical Room participants who were seated in during the experiment.

#### SCR

Results from the repeated measures ANOVA are shown in Table 3. Results revealed a main effect of Condition with significantly increased autonomic response for the spider than the ball, *t*_(30)_ = 5.24, *p* < 0.001, *d* = 1.91. There was also a significant main effect of Distance with greater autonomic response in the Near Approach than Appearance, *t*_(30)_ = −2.58, *p* = 0.015, *d* = 0.94. Since our primary interest was in the Near Approach and the interaction effect between Condition and Distance was marginally significant, we examined the Near Approach revealing a significantly greater SCR response to the spider than the ball *t*_(30)_ = 4.57, *p* < 0.001, *d* = 1.67.

The main effect of Room was not significant (*p* = 0.187) and neither were the between-subjects interactions of Room with Condition and Distance (*p* > 0.05 for each), suggesting that the pattern of SCR between conditions and distance did not depend on the Room where the participants were present.

### Results from experiment 2

We conducted separate repeated measures ANOVAs with Greenhouse-Geisser correction for the pupil and SCR measures and examined the interaction between Condition and Distance. We used paired *t*-tests to test the contrasts. Our primary interest was again the autonomic response in the near approach. The results for the appearance and far approach can be found in the Supplementary Materials.

#### Pupil dilation

Results from the repeated measures ANOVA are shown in Table 3. Results showed a significant main effect of Condition, where mean pupil dilation significantly differed between the stimuli with significantly greater pupil dilation to the spider than to the ball (*p* = 0.004), the spider than the beetle (*p* = 0.004), but not between the beetle and the ball (*p* = 0.139). There was also a significant main effect of Distance. When examining the Near Approach, there was significantly greater pupil dilation to the spider than to the ball (*p* < 0.001), the spider than to the beetle (*p* = 0.004), and the beetle than the ball (*p* = 0.005; see Figure 3b). There was a significant interaction effect between Condition and Distance, which means the pattern of pupil dilation in each condition differed at each distance.

#### SCR

Results from the repeated measures within-subjects ANOVA are shown in Table 3. Results revealed a significant main effect of significantly greater autonomic response to the spider than the ball (*p* = 0.001) and to the beetle than the ball (*p* < 0.001), but not between the spider and the beetle (*p* = 0.081). There was also a significant main effect of Distance. When examining the Near Approach, there was significantly greater SCR to the spider than to the ball (*p* < 0.001), the beetle than the ball (*p* < 0.001), but not the spider than the beetle (*p* = 0.110). There was no significant interaction effect between Condition and Distance.

### Correlations

We combined the spider and the ball data from Experiment 1 and Experiment 2 for both the pupil and SCR responses and calculated difference scores between the spider and the ball (i.e., spider response minus ball response). Results from the correlation cannot make any claims about the nature of the autonomic response—we are only interested in the relationship between the responses from each measure. A correlation analysis revealed that during the Near Approach, the difference scores from the pupil were significantly correlated with the differences scores from the SCR response, *r*_(40)_ = 0.32, *p* = 0.039 (see Figure 3c). All other correlations were non-significant (*p*'s > 0.05).

## General discussion

We effectively and reliably showed physiological differences in autonomic responses in both SCR and pupil dilation measures, with greater autonomic response to the spider than the ball or beetle, regardless of whether participants were in the same virtual and physical environment, or in an entirely separate physical environment. The greater autonomic responses measured by SCR and pupil dilation in response to the spider are not only in the same direction across conditions but were also positively correlated. These differences occurred only when near to the participant in virtual space—a level of immersion that is unique to VR when compared to a monitor screen (Slater et al., 1994; Jones et al., 2008). It is important to note that we did not test distance independent of prior distance, and therefore, it is possible that the current results were blunted by habituation or effects of prior distances. Controlling for this would likely strengthen the current findings. These findings support and extend previous work that has assessed self-reported fear response to spiders in VR (Peperkorn et al., 2016). It is also consistent with previous visual tasks with both children and adults that show attentional bias toward spiders (e.g., Öhman and Mineka, 2001; LoBue, 2010; Devue et al., 2011), presumably due to spiders' recurrent and widespread threat throughout human evolution (Öhman, 1993). For the first time, this study represents that SCR and pupil dilations have been simultaneously recorded and correlated as an autonomic response to spiders, and also such a relationship has been measured in VR.

These findings demonstrate that both SCRs and pupil dilation can be effectively and reliably measured in a VR-based paradigm and appear to tap similar autonomic responses. Furthermore, the autonomic responses are response result of the experimental manipulations and not the physical room context, making reliable remote testing possible. Together, these findings support the notion that a virtual lab with high degree of experimental control can be used using commercially available tools available in people's homes.

The current Virtual Lab overcomes many of the concerns regarding pupil dilation measures (e.g., Aslin, 2012). One challenge has been that pupil size is also determined by low-level stimulus factors like luminance. We have addressed these concerns first through the technical aspect of VR controlling for luminance and a virtually constructed and controlled environment; and second, by correlating the pupil response with another well-established autonomic response. SCR provides a reliable physiological measure of an autonomic response in participants and has been well demonstrated in previous studies as a response to proximal threat, snakes, and spiders (Teghtsoonian and Frost, 1982; Löw et al., 2008). However, SCR is not a measure that can easily be tested in an individual's home without the necessary and expensive equipment, making pupil and eye-tracking measurements a much more convenient tool to tap into physiological responses in a Virtual Lab. One previous experiment showed pupil dilation in a VR decision-making task (Skulmowski et al., 2014); however the VR did not include any head-tracking and they did not control for luminance across conditions. The current study is the first to reliably use pupil dilation measures fully integrated with a VR headset and establish the utility of VR for physiological data collection. Together, we believe this demonstrates a proof-of-concept for a remote Virtual Lab in eye-tracking integration that could change the accessibility of experimental data for researchers.

### Virtual lab: practical considerations

We are just beginning to understand the potential of VR; however, an expanding tech market, previous research studies, and the results of the present study are all positive toward a Virtual Lab, which will be the next step in the future of research. There are, however, some practical issues and caveats to consider when establishing a Virtual Lab.

A current concern for many research fields is a bias in study sample demographics. For example, one meta-analysis revealed that psychology research tends to make conclusions about human nature based on samples taken solely from Western undergraduate students (Henrich et al., 2010). A risk with a Virtual Lab is that research would presumably be tapping a very similar demographic. Indeed, consumer reports suggest that the majority of consumers that purchase a VR system are males (85.7%; Stanton, 2016). However, unlike studies conducted at universities or in research labs, a Virtual Lab is intrinsically connected to an evolving and expanding worldwide tech market. Like other tech markets such as computers and the internet, as the device becomes more main stream the user demographics expand. When compared to the past, online data collection samples today have been shown to be relatively diverse with respect to gender, socioeconomic status, geographic region, and age (Gosling et al., 2004). A possible limitation in the current study was the relatively low number of males when compared to females in the sample. While the distribution of male and female participants is not equal, previous studies have not suggested that gender influences skin conductance and pupil dilation in response to threat (e.g., Partala and Surakka, 2003; Bianchin and Angrilli, 2012; Rosén et al., 2017).

Furthermore, the ease of remote testing with a Virtual Lab serves as a strength for reaching wider demographic samples. With a Virtual Lab, experimenters can remotely test participants across the world while remaining in their own lab, or alternatively bring the VR headset directly to homes or public spaces, all while still maintaining a controlled virtual lab environment. Visual and auditory distractions and differences in testing rooms and luminance would no longer be an issue when testing via VR. Bringing the virtual lab to participants would allow for unique and remote samples that would otherwise be difficult to test due to an inability to physically enter a lab.

While other papers have already engaged in a discussion regarding ethical issues for the implementation of VR in various contexts (e.g., see Whalley, 1995; Brey, 1999), we feel it is important to highlight these concerns here. One concern is the level of immersion and presence that participants may experience, and possible unintended effects on the participants engaging in a virtual environment. VR is considered an “embodied technology” with the ability to modify the feeling of presence (Riva et al., 2016). It can alter our experience of the body and space by altering the very cognitive factors regulating our experience of body and space (for in depth analysis, see Riva et al., 2015). Given VR is an embodied technology, it also opens questions regarding the morality of virtual behavior; further, the behaviors permitted in a virtual environment, which are not socially permitted in real societies, should be acceptable. Finally, there are ethical considerations as to the kinds of data that are collected and stored on a massive scale, and how this data can be secured. Many of these issues are not unique to VR and there are ongoing discussions in many areas of technological development like game design (see Richard Bartle's discussion of “Human Rights and Virtual Worlds”; Bartle, 2004), but nonetheless are important and increasingly relevant issues for researchers.

### Virtual lab: future directions

It is clear that VR headsets are becoming a common addition to households across the world. People play games and engage in virtual experiences regularly in their homes, allowing for the implementation of psychological tests where the information can be fed back to the experimenters and developers on-line. The possibilities of VR are evident by its quick growth into a mainstream tool in the average consumer's home and businesses and institutions, including military, aerospace, construction, automobile industries, entertainment, and popular news and media (Aczél, 2016). The information and data collected in a Virtual Lab experiment can be utilized by researchers and developers for workplace training, software development, marketing and advertising, optimizing game experiences based on arousal and interests of the player/user, and in medicine and treatment. The findings from the current paradigm could be further expanded by examining the physiological response toward spiders depending on levels of anxiety. In the future, it would be very interesting to test the differences between high and low anxious individuals in response to spiders using the virtual lab.

A Virtual Lab provides a useful and robust tool for measuring physiological responses of participants in a controlled virtual environment, where the stimuli can be presented in protection from environmental variation. The current study supports a robust Virtual Lab tool for massive remote testing that combines the strengths of both online testing and lab experiments, which is available through consumer devices, allowing pupil dilation measures in VR. Research on the capabilities and potential of VR is still in its infancy, but both previous and the present results are positive and suggest that a virtual lab is a possible next step in the future of research in a wide variety of fields and industries.

## Ethics statement

This study was carried out in accordance with the recommendations of Uppsala University with written informed consent from all subjects. All subjects gave written informed consent in accordance with the Declaration of Helsinki. The protocol was approved by the Uppsala University Ethics Review Board.

## Author contributions

All authors contributed to the design of the study. NL did programming and created the stimuli. JJ, JR, and GK carried out data collection. JJ, JR, PN, and GK conducted data analyses. JJ and JR wrote the main manuscript text. All authors reviewed and contributed to the final manuscript.

### Conflict of interest statement

NL was employed by company Goodbye Kansas, Visual Effects Studio. The other authors declare that the research was conducted in the absence of any commercial or financial relationships that could be construed as a potential conflict of interest.
