# Supplementary material for: The Immersive Virtual Reality Lab: Possibilities for Remote Experimental Manipulations of Autonomic Activity on a Large Scale
Source: Front Neurosci. 2018 May 8;12:305. doi: 10.3389/fnins.2018.00305 (PMC5951925; doi:10.3389/fnins.2018.00305)
Supplement: Supplementary file 1 [file Data_Sheet_1.DOCX]

Supplemental Materials

**Results**

**Experiment 1**

**Pupil dilation.** When split into three phases, in addition to the Near Approach (*p* < .001), the main effect of Condition was evident in the Far Approach (*p* = .037) with a greater pupil dilation to the ball than to the spider, and there was no main effect in the first Appearance (*p* = .221).

**Range Corrected SCR.** When split into three phases, the main effect of Condition was significant in the Near Approach (*p* = .001), Far Approach (*p* = .048), and the Near Approach (*p* < .001), with greater arousal to the spider than to the ball.

**Experiment 2**

**Pupil dilation.** When split into three phases, the main effect of Condition was evident in the Near Approach (*p* < .001) with greater pupil dilation to the spider than to the ball (*p* < .001), the spider than to the beetle (*p* = .004), and the beetle than the ball (*p =* .005).

**Range Corrected SCR.** When split into three phase, the spider and the ball were significantly different during the Appearance (*p* = .028), Far Approach (*p* = .048), and Near Approach (*p* < .001). The beetle was also significantly different than the ball in the Far Approach (p = .015) and the Near Approach (p < .001).

Figure S1

*An example of the raw data from one participant in Study 1 (10 trials; eye blinks removed and gaps interpolated).Blue (ball) and red (spider) lines mark the start of a trial, and boxed areas mark durations where stimuli were presented.*

*
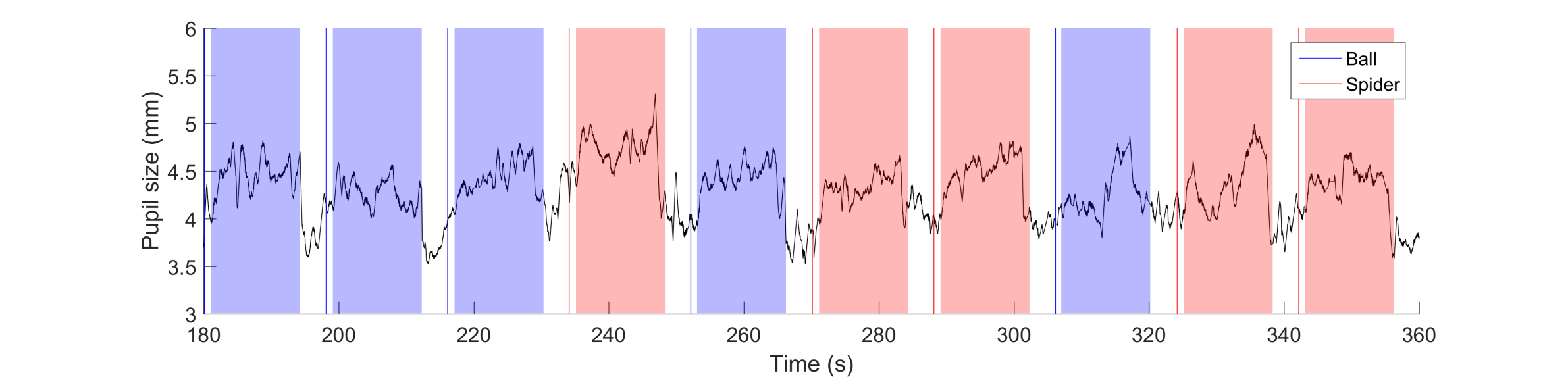
*

Figure S2

*Mean difference scores comparing (a) Spider vs Ball for pupiul dialtion and SCR measures for Far Approch and Near Approach in Study 1, and (b) Spider vs Beetle, Beetle vs Ball, and Spider vs Ball in Study 2.*

*
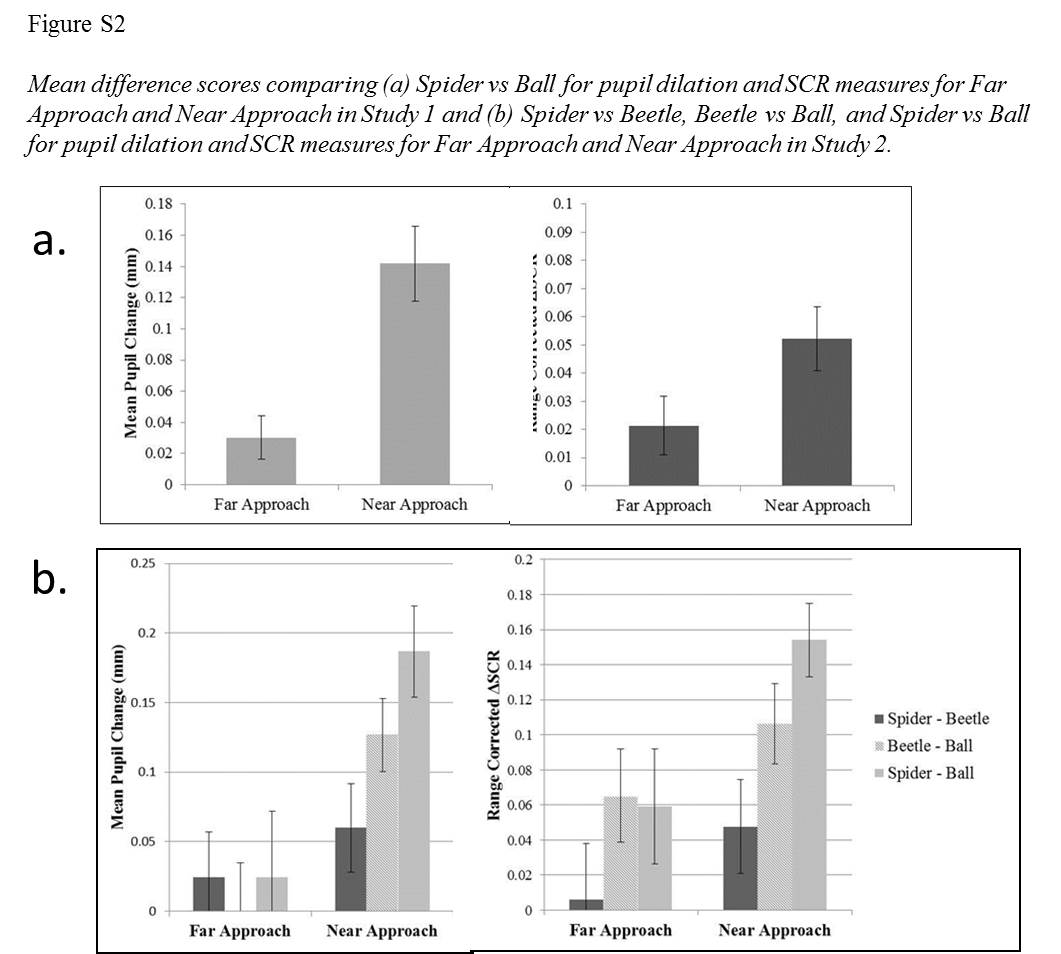
*
